# Quercetin Exhibits Broad-Spectrum Antibiofilm and Antiquorum Sensing Activities Against Gram-Negative Bacteria: In Vitro and In Silico Investigation Targeting Antimicrobial Therapy

**DOI:** 10.1155/cjid/2333207

**Published:** 2025-03-30

**Authors:** Tanvi Shastri, Reem Binsuwaidan, Arif Jamal Siddiqui, Riadh Badraoui, Sadaf Jahan, Nawaf Alshammari, Mohd Adnan, Mitesh Patel

**Affiliations:** ^1^Department of Microbiology, Parul Institute of Applied Sciences, Parul University, Waghodia, Vadodara, Gujarat 391760, India; ^2^Department of Pharmaceutical Sciences, College of Pharmacy, Princess Nourah bint Abdulrahman University, P.O. Box 84428, Riyadh 11671, Saudi Arabia; ^3^Department of Biology, College of Science, University of Ha'il, P.O. Box 2440, Ha'il, Saudi Arabia; ^4^Department of Medical Laboratory Sciences, College of Applied Medical Sciences, Majmaah University, Majmaah 11952, Saudi Arabia; ^5^Research and Development Cell (RDC), Parul University, Waghodia, Vadodara, Gujarat 391760, India; ^6^Department of Biotechnology, Parul Institute of Applied Sciences, Parul University, Waghodia, Vadodara, Gujarat 391760, India

**Keywords:** antimicrobial therapy, biofilms, molecular docking, molecular dynamics, quercetin, quorum sensing

## Abstract

Quercetin (QC), a flavonoid abundant in fruits and vegetables, has garnered attention for its potential therapeutic properties. In this study, we investigated the antibiofilm and antiquorum sensing (QS) activities of QC against Gram-negative bacteria both in vitro and in silico. The findings of this study demonstrate MIC values of 125 μg/mL for *Chromobacterium violaceum*, 250 μg/mL for *Pseudomonas aeruginosa*, and 500 μg/mL for *Serratia marcescens*, indicating its antibacterial potential abilities. QS-mediated production of violacein and prodigiosin was significantly inhibited in a dose-dependent manner at sub-MIC concentrations. Additionally, a dose-dependent reduction in the virulence factors of *P. aeruginosa*, including production of pyocyanin, pyoverdine, and rhamnolipid, was noted with QC. Biofilm formation decreased by 66.40%, 59.28%, and 63.70% at the highest sub-MIC for *C. violaceum*, *P. aeruginosa*, and *S. marcescens*, respectively. Furthermore, swimming motility and exopolysaccharide (EPS) production were also reduced in the presence of QC. Additionally, molecular docking and molecular dynamics simulations elucidate the binding interactions between QC and key molecular targets (LasI, LasR, PilY1, LasA, PilT, CviR, CviR′, PqsR, RhlR, and PigG) involved in biofilm formation and QS pathways. Our results indicated that the antibiofilm and anti-QS sensing activities of QC may be attributed to its ability to interfere with critical signaling molecules and regulatory proteins. Overall, this study highlights QC as a promising natural compound for combating biofilm-associated infections caused by Gram-negative bacteria. The multifaceted antimicrobial mechanisms of QC underscore its potential as a therapeutic agent for the treatment of biofilm-related infections, providing the way for further exploration, and development of QC-based strategies in antimicrobial therapy.

## 1. Introduction

Biofilms represent bacterial associations embedded within an extracellular matrix comprising exopolysaccharides (EPSs), proteins, and various micromolecules such as DNA [1]. These associations can thrive on both living and nonliving surfaces [2]. Numerous pathogenic microorganisms within host organisms can form biofilms, contributing to persistent infections that may lead to chronic illnesses and increasing antibiotic resistance [3]. In the context of wound colonization, biofilms play a dual role by safeguarding pathogens from the defense mechanism of the host and hindering antibiotic sensitivity, thereby protecting from the overall process of wound healing [4–6]. Addressing the challenges posed by biofilm-associated infestations requires the development of innovative antibiofilm agents [7–9]. Consequently, there is an immediate need for antibiotics designed specifically to target biofilms, constituting a promising strategy in novel drug design [10]. Approaches to combat biofilms involve targeting microbe-specific proteins, and enzymes or disrupting adhesion pathways to prevent the formation of biofilms [11, 12]. The mechanisms employed by antibiofilm agents encompass hindering attachment with the surface of bacteria, disrupting the communicating mechanism, modulating nucleotide second messenger signaling, inhibiting biodiversification of genome of bacteria, and inducing dispersal of biofilm on different surfaces [11–15].

Recognition of natural compounds specially derived from plants as a valuable reservoir has emerged, offering the prospect of developing antimicrobial agents with novel mechanisms [1, 2]. The incorporation of bioactive plant-based products into feed and foods holds promise for enhancing individual health [16, 17]. In this context, polyphenolic compounds, specifically flavonoids, have garnered attention for their antibacterial properties manifested through diverse mechanisms. These flavonoids exhibit the capability to diminish the permeability of membrane, pathogenicity, porin of cell membranes, adhesion processes, and development of biofilm that all are integral factors for the growth of bacteria [18–22].

Quercetin, belonging to the flavonoid class which is widespread in various plant parts, is characterized by three benzene rings and five hydroxyl groups [23]. The highest concentrations are found in onions, and asparagus, while several vegetables such as, peas, broccoli, green peppers, and tomatoes, contain lower amounts. There are certain fruits that contain the most quercetin, such as apples, cherries, and blueberries [24]. Small and large intestines hydrolyze, and absorb quercetin glycosides, which have a short half-life in the blood [23]. Physiological processes in targeted tissues utilize the deconjugated aglycone from conjugated metabolites in the plasma or other tissues [25, 26]. Due to its lack of sugar moieties, this versatile compound interacts with different targets of the cells, inhibiting multiple pathways [27]. Quercetin has the potential to be used as a drug molecule due to its ability to inhibit key enzymes [28–30].

Quercetin displays a wide range of pharmacological activities, such as antioxidant, anticancer, antiviral, antimicrobial, neuroprotective, anti-inflammatory, cardiovascular, and anti-obesity effects [30, 31]. Recently, the United States Food and Drug Organization has granted quercetin the generally recognized as safe (GRAS) status [29]. The antimicrobial potential of quercetin has been extensively reported, positioning it as a promising therapeutic option against various pathogenic microorganisms [31]. The escalating global issue of multidrug resistance (MDR) in bacteria, restricting from the persistent use of antibiotics, presents a substantial challenge for the pharmaceutical industry [32]. The scientific community has increasingly turned its attention to natural biological active compounds derived from various natural resources because of their advantageous properties on human health [24].

To address the rising concern of MDR strains, there is an increasing focus on understanding the antimicrobial mechanisms of phytochemicals for effective drug development. Previous investigations into the pharmaceutical properties of quercetin have highlighted its effectiveness as a powerful natural antimicrobial agent toward different infectious microbes [23–26]. Consequently, the present study focused on evaluating antibacterial, antibiofilm, and antiquorum sensing (anti-QS) properties of quercetin against different bacterial pathogens via different in silico, and in vitro approaches. Based on the findings of this study, impact of quercetin on different QS-related phenomena of bacteria, which provides valuable insights. These results enhance the scientific comprehension of effectiveness of quercetin in combating microbial infections and highlight its potential use as an antimicrobial agent in medical applications.

## 2. Materials and Methods

### 2.1. Antibacterial Assays

#### 2.1.1. Bacterial Strains

A comprehensive investigation into the antibacterial efficacy of QC was conducted against Gram-negative pathogenic bacteria including *Pseudomonas aeruginosa*, *Serratia marcescens*, and *Chromobacterium violaceum*. Bacterial strains were procured from the Microbial Type Culture Collection (MTCC) in Chandigarh, India, and were grown on Muller–Hinton agar (MHA) plates. A fresh medium was inoculated with a single bacterial colony and allowed to grow overnight at 37°C. Adjusting the turbidity of the culture to match the 0.5 McFarland standard (10^8^ CFU/mL) was achieved using sterile saline solution.

### 2.2. Agar Cup Diffusion Assay

The antibacterial activity of QC was assessed using the agar cup/well diffusion method on MHA plates. Individual bacterial culture (100 μL each) was spread on the plates, and wells were made using gel puncture. Subsequently, 50 μL of QC (1000 μg/mL) was introduced into each well and the plates were incubated at 37°C for 24 h. The following day, the zones of inhibition were measured. As a positive control, streptomycin (1000 μg/mL) was employed, while 10% dimethyl sulfoxide (DMSO) (Hi-media, India) served as the negative control for the assay [33].

### 2.3. Determination of Minimum Inhibitory Concentration (MIC)

For the determination of MIC values of QC, a broth dilution method was employed following the procedures outlined in CLSI (2014) [34]. QC was subjected to double-fold derail dilution in Mueller–Hinton broth (MHB) containing bacterial culture (10^8^ CFU/mL, 0.5 McFarland standard). Concentrations of QC ranged from 1000 to 1.95 μg/mL. Incubation was performed at 37°C for 24 h. Based on the results of the QC, MIC was determined to be the lowest concentration that prevented any growth in the tubes, confirmed by measuring the turbidity via colorimeter before and after incubation. As positive controls, streptomycin was used, MHB + DMSO (10%) was used as vehicle controls, and MHB alone was used as sterility controls.

### 2.4. Determination of Minimum Bactericidal Concentration (MBC)

The determination of MBC was carried out followed by the MIC assay. From wells displaying no apparent growth, 5 μL of the sample was streaked onto MHA plates. These plates were then incubated at 37°C for 18–24 h. The MBC value was subsequently noted as the concentration at which the minimal growth or colony formation of bacteria was observed [35].

### 2.5. Assessment of Violacein Pigment Production in *C. violaceum*

According to a previously described procedure, spectrophotometric measurements were conducted to assess the relative quantity of violacein pigment production [36]. The overnight-grown culture of *C. violaceum* was inoculated in LB broth both in the absence and presence of QC. The inhibitory effect of QC was evaluated at sub-MIC equivalent to 1/8, 1/4, and 1/2 MIC. The bacterial culture was allowed to grow at 30°C for 24 h. Incubation was followed by centrifugation at 10,000 rpm for 5 min with 1 mL of culture from each tube. Following vigorous vortexing of the pellet, 1 mL of DMSO (Hi-media, India) was used to dissolve the violacein pigment. To settle down the cells, the mixture was centrifuged again. With a UV-2600 spectrophotometer (Shimadzu, Japan), the supernatant at 585 nm was measured against DMSO to determine its optical density.

### 2.6. Determination of Virulence Factors of *S. marcescens*

The standard procedure outlined by [37] was employed to determine prodigiosin pigment production. The *S. marcescens* MTCC 97 strain was cultivated in LB broth with or without sub-MIC levels of QC for 18 h at 30°C under shaking conditions at 120 rpm. As a result of incubation, centrifugation was used to collect the cell pellet, which was then redissolved in 1 mL of acidified ethanol (96 mL ethanol + 4 mL 1 M HCl). A spectrophotometer (UV-2600, Shimadzu, Japan) was used to measure the absorbance of the supernatant (containing prodigiosin) at 534 nm following centrifugation at 13,000 rpm for 5 min to precipitate cell debris.

(1) $$\begin{array}{c} \%\text{Prodigiosin inhibition}=\left(\frac{\text{OD control}–\text{OD test}}{\text{OD control}}\right)\times 100. \end{array}$$

### 2.7. Quantitative Analysis of Pyocyanin Production in *P. aeruginosa*

The method described by [38] was used to quantify the production of pyocyanin by *P. aeruginosa* culture supernatants. The extraction process was carried out by extracting 1.5 mL of untreated or QC-treated (sub-MICs) *P. aeruginosa* supernatant with 3 mL of chloroform, followed by a re-extraction with 0.2 M HCl (700 μL). The resulting solution was then utilized to measure absorbance at 595 nm. The inhibition of pyocyanin production was calculated via following formula:

(2) $$\begin{array}{c} \%\text{Pyocyanin inhibition}=\left(\frac{\text{OD control}–\text{OD test}}{\text{OD control}}\right)\times 100. \end{array}$$

### 2.8. Assessment of Pyoverdine

In accordance with the established protocol, the analysis of pyoverdine levels was conducted according to [39]. *P. aeruginosa* was cultured overnight at 37°C, both without and with QC at sub-MIC concentration. A centrifugation step was carried out to obtain a cell-free supernatant. Subsequently, 900 μL of 50 mM Tris–HCl (pH-7.4) was combined with 100 μL of the culture supernatant. The fluorescence emission signal of the sample was measured at 460 nm using a multimode microplate reader (Biotek, USA).

### 2.9. Rhamnolipid Quantification

*P. aeruginosa* was cultured in LB broth with and without QC at subconcentrations of MIC and then incubated at 37°C for 24 h. The extraction of rhamnolipids was carried out through the ethyl acetate evaporation method. The extracted rhamnolipids were dissolved in chloroform and their quantity produced by *P. aeruginosa* was measured using a modified version of the [40] method. After extraction, 200 μL of a freshly prepared 0.025% methylene blue solution was added to 2 mL of the rhamnolipid solution. After vortexing vigorously for 5 min, the mixture was left at room temperature for 15 min. In the next step, the chloroform layer was transferred into new tubes with 500 μL of 0.2 N HCl, mixed thoroughly, and allowed to stand for 10 min to allow the phase separation by working at room temperature. Lastly, 200 μL of acidic phase containing methylene blue complex was spectrophotometrically measured at 638 nm with 0.2 N HCl as a blank.

### 2.10. Determination of Antibiofilm Activity

To evaluate the antibiofilm efficacy of QC, glass test tubes were employed as the hydrophilic surface, following the methodology outlined by [41]. In brief, sterilized MHB and 1% glucose medium (3 mL) were added to tubes having 250 μL of bacterial culture and 250 μL of QC at sub-MIC levels. Contents of the tubes were thoroughly mixed and incubated at 37°C and 120 rpm for 72 h. At the end of incubation, the tube contents were discarded and rinsed with PBS to eliminate unattached bacteria. A 0.1% crystal violet stain was used to stain the formed biofilm on the surfaces of the tubes. Following the removal of unbound dye using PBS, the dye absorbed by the biofilm was extracted with acetic acid, and its density was measured using a spectrophotometer at 595 nm. *Pseudomonas aeruginosa*, *Serratia marcescens*, and *Chromobacterium violaceum* were individually used as controls for biofilm growth on MHB medium. Based on the following formula, the percentage of biofilm inhibition was calculated:

(3) $$\begin{array}{c} \left(\frac{\text{OD control}–\text{OD test}}{\text{OD control}}\right)\times 100. \end{array}$$

### 2.11. Determination of the Swarming Motility

The swarming agar plate (1% peptone, 0.5% NaCl, and 0.3% agar) with or without sub-MIC (1/2 MIC) of QC was inoculated with 50 μL of the active culture of *P. aeruginosa*, *S. marcescens*, and *C. violaceum*. Overnight incubation was performed at 30°C. After the incubation period, the presence of a swarming zone was observed in both the control and treatment plates [42].

### 2.12. Extraction and Quantification of EPS

EPS production by bacterial strain after QC treatment was estimated using a modified method from [43]. Briefly, cultures were grown in LB broth in presence or absence of QC at sub-MIC levels at 37°C for 18 h. Cell pellets were resuspended in a high salt buffer after centrifuging at 10,000 rpm for 10 min postincubation. A second centrifugation at 10,000 rpm for 15 min was performed on these suspensions. Precipitating the released EPS was achieved by treating the supernatant with 95% ethanol. As an equal volume of milli-Q water and 5% ice-cold phenol were added to the precipitated EPS, the EPS was dissolved in it. The reaction mixture was colored red by adding concentrated H_2_SO_4_ at a ratio of 2:5. A spectrophotometer was used to measure the absorbance at 490 nm of EPS produced.

### 2.13. Molecular Docking Analysis

The mechanism behind antibiofilm and anti-QS activities of QC was explored using molecular docking with AutoDock Vina [44]. The structure of QC [CID: 5280343] was downloaded from PubChem in .sdf form and converted to.pdb form using Open Babel V2.4.1. Structural optimization was performed by minimizing energy using Avogadro, applying the MMFF94 force field, and a 5000-step steepest descent algorithm. A 3D crystal structure of each target protein was obtained from the Protein Data Bank. In the case of RhlR, the 3D crystal structures were obtained from the SWISS-MODEL Repository (ID: P54292), since it was not available in the Protein Data Bank. AutoDock Tools 1.5.7 was used to prepare these structures for docking, where water molecules were removed, and polar hydrogen atoms, and Kollman partial charges were added. In order to perform docking analysis, the proteins were saved in .pdbqt format. PyMOL and Discovery Studio were used to analyze docked complexes [45].

### 2.14. Molecular Dynamics Simulation

Molecular dynamics (MD) is a technique used to simulate the physical motions, including the stability and flexibility of atoms, and molecules. It is widely used by biologists to examine different solute environments [46, 47]. The MD simulations in this study were conducted using the GROMACS package with the CHARMM36m force field [48]. Ligand topology files and force field parameters were generated using the SwissParam web server. Initial system minimization was carried out in a vacuum for 1500 steps via steepest descent algorithm until the maximum force was reduced below 1000.0 kJ/mol/nm. The protein–ligand complex was then solvated in an orthorhombic periodic box with dimensions of 10 Å × 10 Å × 10 Å, using the TIP3*p* water model. The system was neutralized with Na^+^ and Cl^−^ counterions at a concentration of 0.15M. Equilibration steps were performed in both NVT and NPT ensembles for 0.1 ns each, using the leap-frog algorithm. During the MD simulation, the temperature and pressure were maintained at 300 K and 1.01325 bar, respectively. The production of MD run lasted for 100 ns on the solvated protein–ligand complex. After the MD simulation, periodic boundary conditions were removed from the trajectory file. The flexibility and stability of the protein–ligand complex were analyzed using XMGrace software. Key metrics such as root-mean-square deviation (RMSD), root-mean-square fluctuation (RMSF), the radius of gyration (Rg), hydrogen bonds, and solvent-accessible surface area (SASA) were calculated via the GROMACS program to assess the stability and compactness of the structure [49].

### 2.15. Statistical Analysis

The data are reported as the mean ± standard deviation, based on the number of experiments conducted. To determine the significance of the results, we used an ordinary one-way ANOVA, followed by Bonferroni's multiple comparisons test, with a significance threshold set at *p* < 0.05. All analyses were performed using GraphPad Prism software Version 8.0 (GraphPad Software, USA).

## 3. Results

### 3.1. Antibacterial Potential of QC

**Figure 1:**
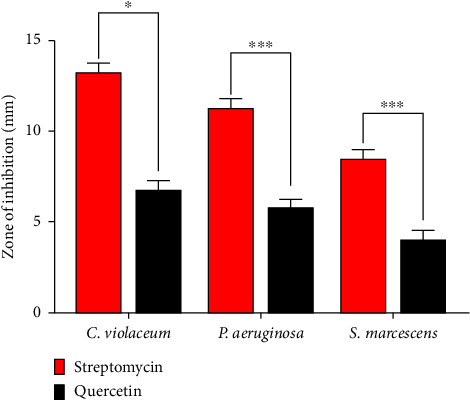
Evaluation of antibacterial efficacy of QC and streptomycin against various bacterial pathogens. Values are represented as the mean ± SD of three independent experiments. Significance; ns > 0.05, ^∗^*p* < 0.05, ^∗∗^*p* < 0.005, and ^∗∗∗^*p* < 0.0005 with respect to each other.

QC exhibited antibacterial activity toward the tested bacterial strains via agar well diffusion assays. Among the strains, *P. aeruginosa* exhibited the largest zone of inhibition, followed by *C. violaceum*, and *S. marcescens* (Figure 1). The MIC values of QC, determined through the broth microdilution method, were 125 μg/mL for *C. violaceum*, 250 μg/mL for *P. aeruginosa*, and 500 μg/mL for *S. marcescens*. The MBC values were 500 μg/mL for *C. violaceum* and 1000 μg/mL for both *P. aeruginosa* and *S. marcescens*. The efficacy of QC in inhibiting formation of biofilm and QS-regulated virulence factors was assessed at sub-MIC concentrations (1/2, 1/4, and 1/8 MIC).

### 3.2. Inhibition of Virulence Factors of *C. violaceum*

**Figure 2:**
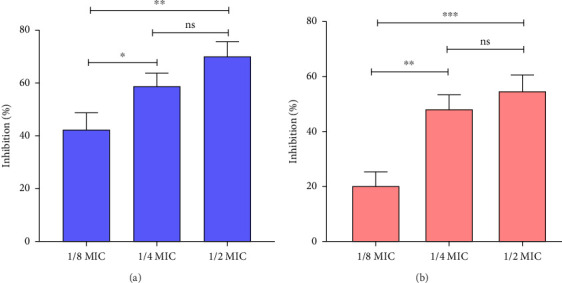
(a) Quantitative analysis of violacein inhibition in *C. violaceum* using QC and (b) quantitative analysis of prodigiosin inhibition in *S. marcescens* using QC. Values are represented as the mean ± SD of three independent experiments. Significance; ns > 0.05, ⁣^*∗*^*p* < 0.05, ⁣^*∗∗*^*p* < 0.005, ⁣^*∗∗∗*^*p* < 0.0005 with respect to each other.

The initial anti-QS activity of QC was assessed by examining its impact on pigment production in *C. violaceum*, which is regulated via quorum sensing. A decrease in pigment production indicates potential anti-QS properties. Treatment with QC at 1/2, 1/4, and 1/8 MIC resulted in reductions of violacein synthesis by 69.88%, 58.87%, and 42.38%, respectively (Figure 2(a)). These results strongly suggest that QC possesses anti-QS activity.

### 3.3. Inhibition of Virulence Factors of *S. marcescens*

The QC was also determined for its anti-QS assay against *S. marcescens* to evaluate its potential broad-spectrum QS inhibition. *S. marcescens* produces the QS-regulated pigment prodigiosin.  Figure 2(b) illustrates that various sub-MIC concentrations of QC reduced prodigiosin production in *S. marcescens*. Specifically, at concentrations of 1/2, 1/4, and 1/8 MIC, QC inhibited prodigiosin production by 54.58%, 48.20%, and 20.31%, respectively. These results indicate capability of QC to inhibit QS in *S. marcescens*.

### 3.4. Inhibition of Virulence Factors of *P. aeruginosa*

**Figure 3:**
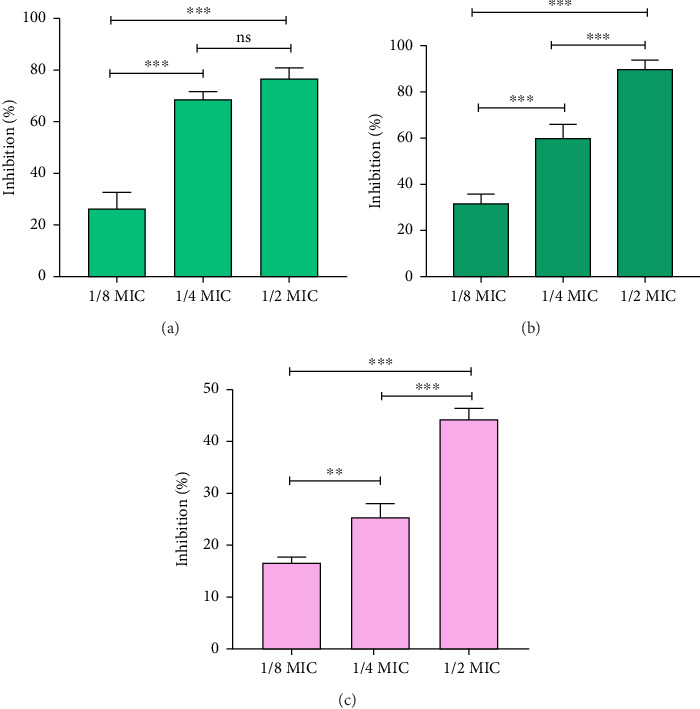
(a) Quantitative analysis of pyocyanin inhibition in *P. aeruginosa* using QC, (b) quantitative analysis of pyoverdine inhibition in *P. aeruginosa* using QC, and (c) quantitative analysis of rhamnolipid inhibition in *P. aeruginosa* using QC. Values are represented as the mean ± SD of three independent experiments. Significance; ns > 0.05, ⁣^*∗*^*p* < 0.05, ⁣^*∗∗*^*p* < 0.005, ⁣^*∗∗∗*^*p* < 0.0005 with respect to each other.

Impact of QC on different QS-mediated virulence factors of *P. aeruginosa* was investigated, particularly its effect on pigment production including pyocyanin, and pyoverdine. Treatment with sub-MIC concentrations of QC (1/2, 1/4, and 1/8 MIC) resulted in gradual reductions in pyocyanin production by 76.65%, 68.71%, and 26.30%, respectively (Figure 3(a)). Furthermore, QC inhibited pyoverdine synthesis by *P. aeruginosa* in a concentration-dependent manner, with reductions of 90.11%, 59.80%, and 31.68% at corresponding sub-MIC concentrations (Figure 3(b)). Additionally, QC also led to a reduction in rhamnolipid production, crucial for biofilm structure and bacterial adhesion to surfaces, regulated by RhlR-RhlI QS in *P. aeruginosa*. Rhamnolipid production decreased by 44.22%, 25.39%, and 16.75% with sub-MIC concentrations of QC (Figure 3(c)). These findings highlight the potential of QC to attenuate *P. aeruginosa* virulence through QS inhibition.

### 3.5. Effect of QC on Biofilm Inhibition

**Figure 4:**
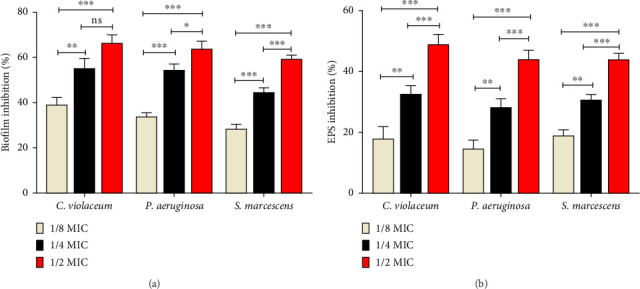
(a) Quantitative analysis of inhibition of biofilm using QC and (b) quantitative analysis of inhibition of EPS using QC. Values are represented as the mean ± SD of three independent experiments. Significance; ns > 0.05, ⁣^*∗*^*p* < 0.05, ⁣^*∗∗*^*p* < 0.005, ⁣^*∗∗∗*^*p* < 0.0005 with respect to each other.

The effect of QC on formation of biofilm, regulated by AI-mediated QS, was investigated for all three tested bacteria. In Figure 4(a), the inhibition percentages of biofilm formation are presented. For *C. violaceum*, treatment with 1/2, 1/4, and 1/8 MIC concentrations of QC resulted in inhibition of biofilm formation by 66.40%, 55.25%, and 39.14%, respectively. In the case of *P. aeruginosa*, biofilm formation was inhibited by 63.70%, 53.65%, and 33.90% at sub-MIC concentrations of QC. Similarly, *S. marcescens* biofilms decreased by 59.28%, 44.53%, and 28.35%, respectively, in the presence of sub-MICs of QC. These findings suggest potential of QC to inhibit biofilm formation across various bacterial species, possibly through interference with QS mechanisms.

### 3.6. Inhibition of EPS Production

It was further observed that treatment with QC led to a reduction in EPS production which is vital for biofilm formation, stability, and functionality. For *C. violaceum*, EPS production decreased by 48.88%, 32.67%, and 16.92%. In the case of *P. aeruginosa*, EPS production decreased by 44.90%, 28.34%, and 14.70%. Similarly, for *S. marcescens*, EPS production decreased by 43.51%, 30.77%, and 18.86% (Figure 4(b)). These findings suggest that QC treatment disrupts EPS production across different bacterial species, which may contribute to the inhibition of biofilm formation, and compromise the integrity, and resilience of existing biofilms.

### 3.7. Inhibition of Swarming Motility

**Figure 5:**
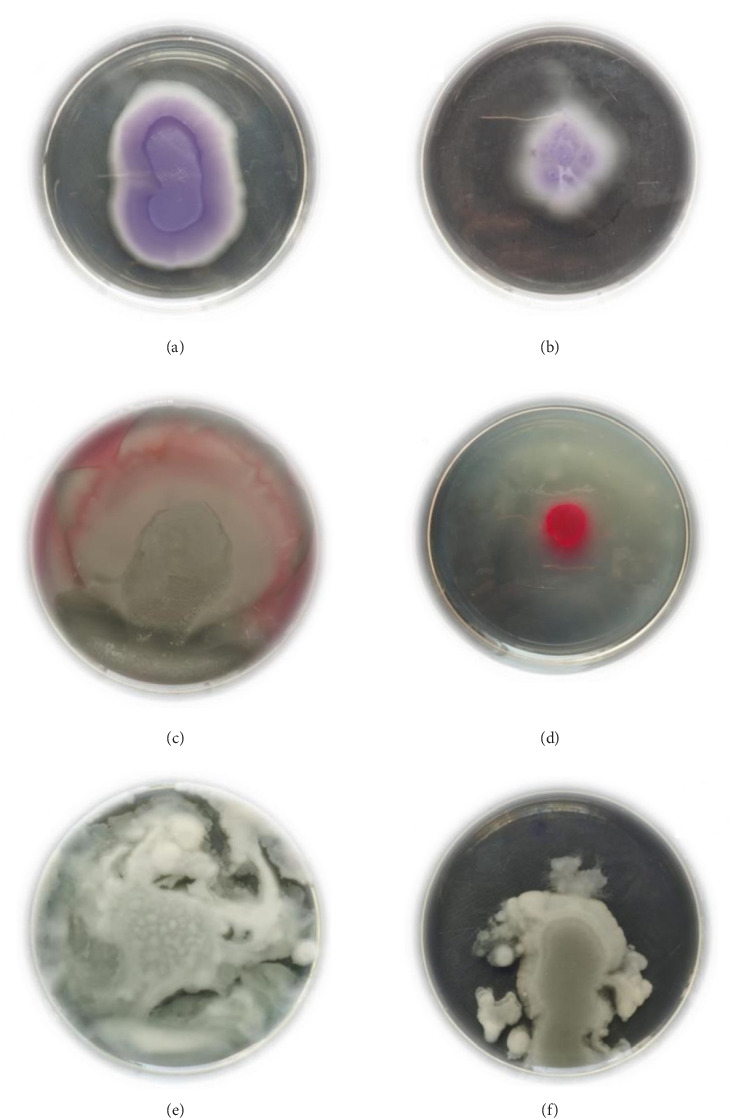
Swarming motility assay (a) control of *C. violaceum*, (b) treatment of *C. violaceum* with 1/2 MIC of QC, (c) control of *S. marcescens*, (d) treatment of *S. marcescens* with 1/2 MIC of QC, (e) control of *P. aeruginosa*, and (f) treatment of *P. aeruginosa* with 1/2 MIC of QC.

The impact of QC on swarming motility of *S. marcescens*, *P. aeruginosa*, and *C. violaceum* is presented in Figure 5. In comparison with controls, QC-treated samples exhibited significantly reduced swarming motility across all three bacterial species.

### 3.8. Molecular Docking

**Figure 6:**
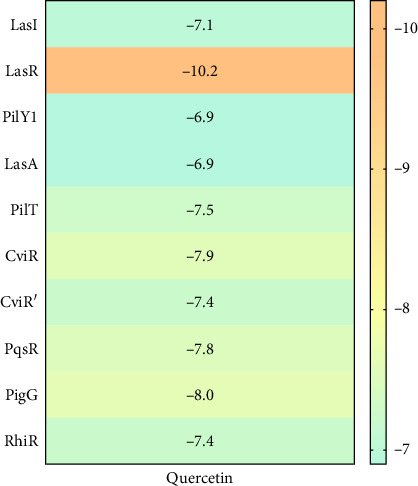
Heatmap of top binding affinities of the QC against biofilm and QS targets.

**Figure 7:**
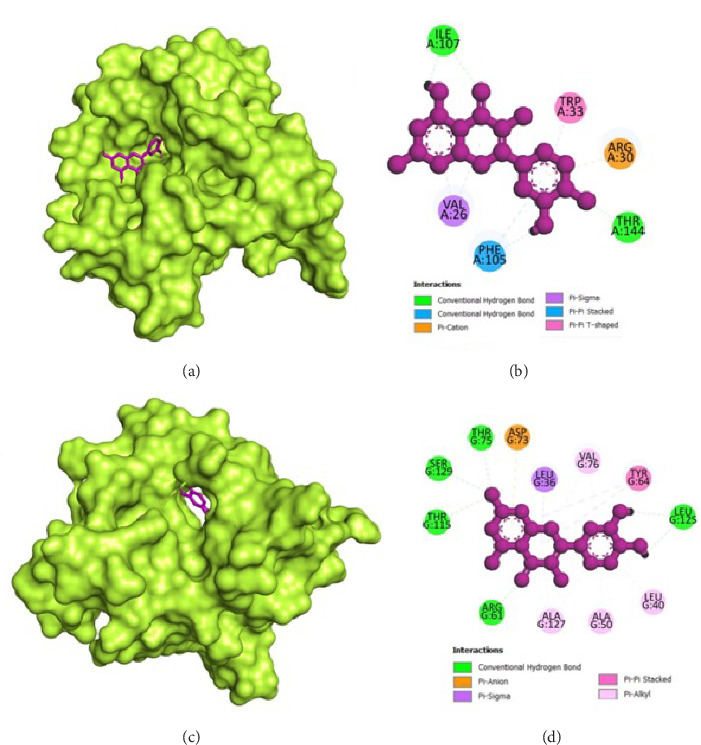
Illustration of the binding interactions between QC with biofilm and QS targets. (a) Three-dimensional hydrophobicity surface representation of QC with the LasI protein, (b) two-dimensional interactions between QC and the LasI protein, (c) three-dimensional hydrophobicity surface representation of QC with the LasR protein, and (d) two-dimensional interactions between QC and the LasR protein.

**Figure 8:**
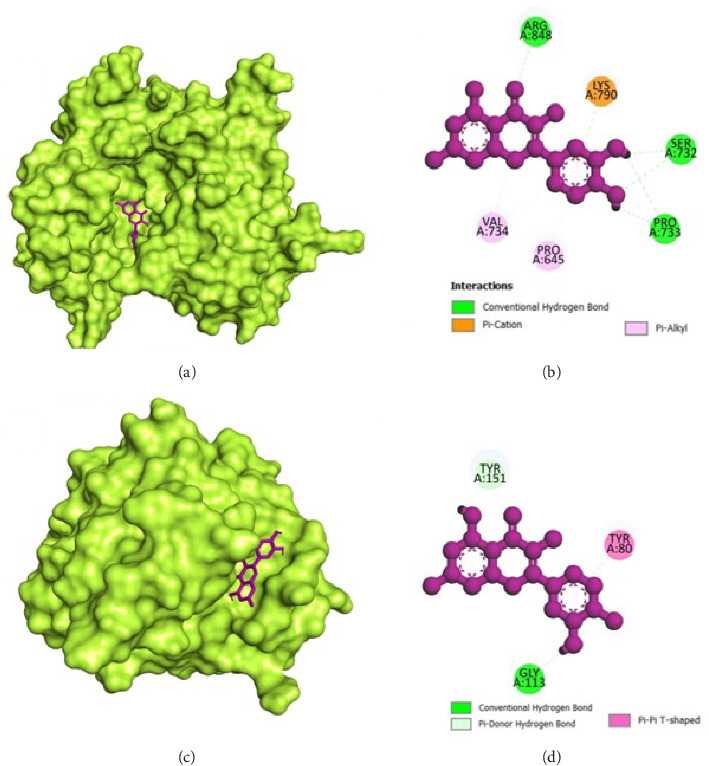
Illustration of the binding interactions between QC with biofilm and QS targets. (a) Three-dimensional hydrophobicity surface representation of QC with the PilY1 protein, (b) two-dimensional interactions between QC and the PilY1 protein, (c) three-dimensional hydrophobicity surface representation of QC with the LasA protein, and (d) two-dimensional interactions between QC and the LasA protein.

**Figure 9:**
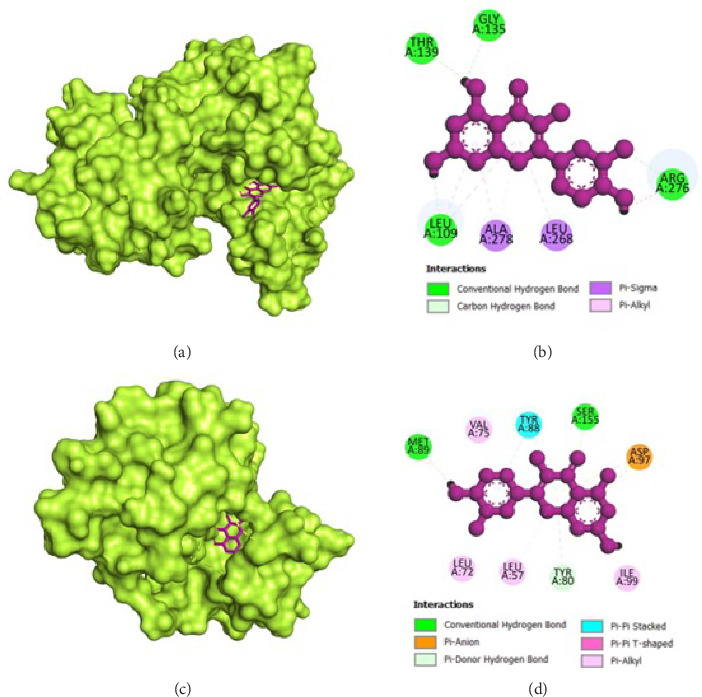
Illustration of the binding interactions between QC with biofilm and QS targets. (a) three-dimensional hydrophobicity surface representation of QC with the PilT protein, (b) two-dimensional interactions between QC and the PilT protein, (c) three-dimensional hydrophobicity surface representation of QC with the CviR protein, and (d) two-dimensional interactions between QC and the CviR protein.

**Figure 10:**
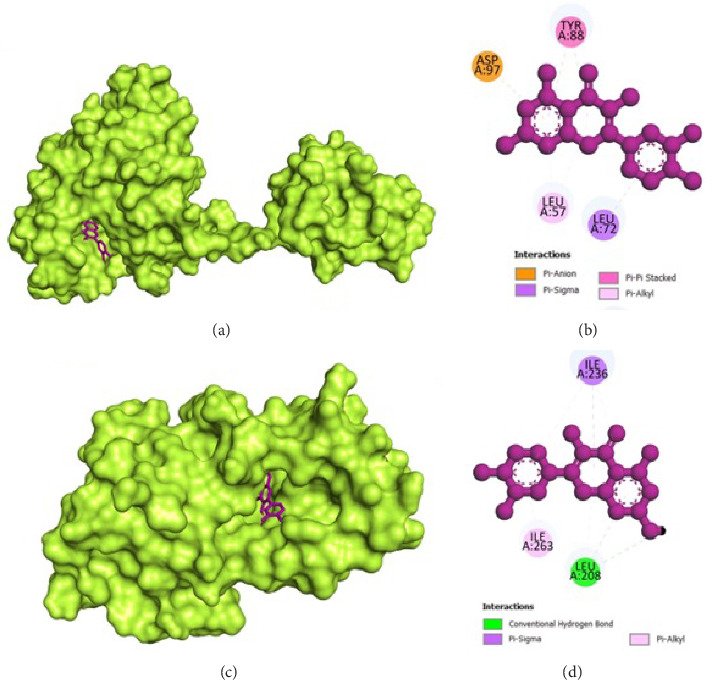
Illustration of the binding interactions between QC with biofilm and QS targets. (a) Three-dimensional hydrophobicity surface representation of QC with the CviR′ protein, (b) two-dimensional interactions between QC and the CviR′ protein, (c) three-dimensional hydrophobicity surface representation of QC with the PqsR protein, and (d) two-dimensional interactions between QC and the PqsR protein.

**Figure 11:**
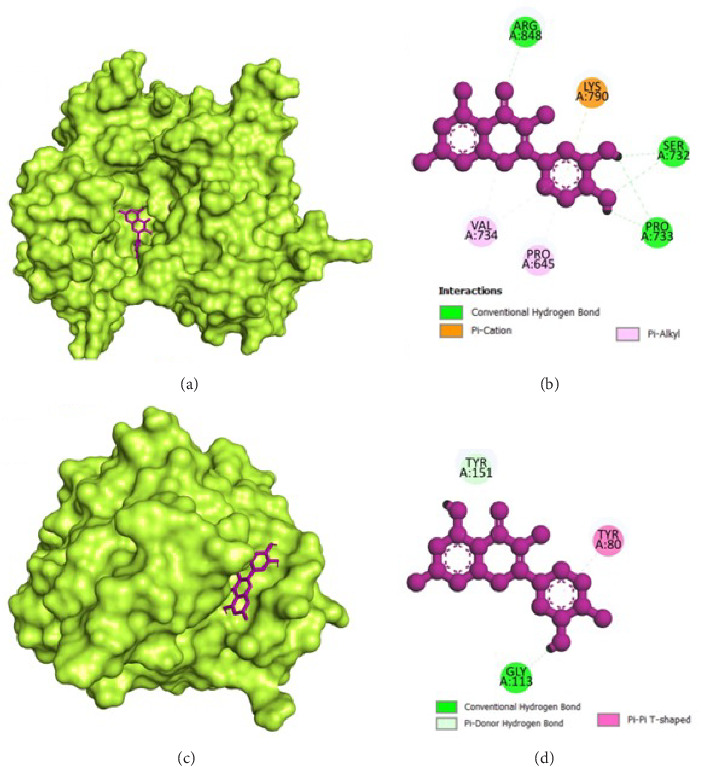
Illustration of the binding interactions between QC with biofilm and QS targets. (a) Three-dimensional hydrophobicity surface representation of QC with the PigG protein, (b) two-dimensional interactions between QC and the PigG protein, (c) three-dimensional hydrophobicity surface representation of QC with the RhiR protein, and (d) two-dimensional interactions between QC and the RhiR protein.

The molecular docking analysis results are represented which aimed to investigate the antivirulence potential of QC by examining its interactions through proteins associated with QS and biofilm formation. QC displayed varying binding affinities with the tested proteins, as indicated by the lowest binding energies. QC exhibited lowest binding energy with LasI (−7.1 kcal/mol) with four conventional hydrogen bond (ILE107, THR144, PHE105, ILE107), one pi-cation bond (ARG30), two pi-sigma bond (2⁣^∗^VAL26), one pi-pi stacked bonds (PHE105), and one pi-pi T-shaped bond (TRP33), toward LasR (−10.2 kcal/mol) with six conventional hydrogen bond (ARG61, THR75, THR115, SER129, and 2⁣^∗^LEU125), one pi-anion bond (ASP73), one pi-sigma bond (LEU36), two pi-pi stacked bonds (2⁣^∗^TYR64), and eight pi-alkyl bonds (2⁣^∗^VAL76, 2⁣^∗^ALA127, LEU36, ALA127, LEU40, and ALA50), toward PilY1 (−6.9 kcal/mol) with six conventional hydrogen bond (2⁣^∗^SER732, 2⁣^∗^ARG848, and 2⁣^∗^PRO733), one pi-cation bond (LYS790), and four pi-alkyl bonds (2⁣^∗^VAL734, PRO645, and LYS790), toward LasA (−6.9 kcal/mol) with one conventional hydrogen bond (GLY113), two pi-donor hydrogen bond (2⁣^∗^TYR151), and one pi-pi T-shaped bond (TYR80), toward PilT (−7.5 kcal/mol) with five conventional hydrogen bond (THR139, 2⁣^∗^ARG276, LEU109, GLY135), one carbon hydrogen bond (GLY135), four pi-sigma bond (2⁣^∗^LEU109, LEU278, and ALA278), and two pi-alkyl bonds (ALA278 and ARG276), toward CviR (−7.9 kcal/mol) with two conventional hydrogen bond (SER155 and MET89), two pi-anion bonds (2⁣^∗^ASP97), one pi-anion bond (ASP97), one pi-donor hydrogen bond (TYR80), two pi-pi stacked bonds (2⁣^∗^TYR88), two pi-pi T-shaped bonds (2⁣^∗^TYR80), and four pi-alkyl bonds (LEU57, ILE99, LEU72, and VAL75), toward CviR' (−7.4 kcal/mol) with one pi-anion bond (ASP97), one pi-sigma bond (LEU72), two pi-pi stacked bonds (2⁣^∗^TYR88), and two pi-alkyl bonds (2⁣^∗^LEU57), toward PqsR (−7.8 kcal/mol) with one conventional hydrogen bond (LEU208), two pi-sigma bonds (LEU208 and ILE236), and four pi-alkyl bonds (LEU208, 2⁣^∗^ILE236, and ILE263), toward RhlR (−7.4 kcal/mol) with three conventional hydrogen bond (VAL47 and 2⁣^∗^TRP68), two pi-anion bonds (2⁣^∗^ASP81), two pi-sigma bonds (VAL60 and ILE84), two pi-pi stacked bonds (TYR72 and TRP96), and one pi-alkyl bond (ALA83), and toward PigG (−8 kcal/mol) with two conventional hydrogen bond (LEU264 and GLY262), one pi-cation bond (LYS17), two pi-anion bonds (2⁣^∗^GLU113), and one pi-alkyl bond (LEU264). The heatmap of top binding affinities of the QC against biofilm and QS targets (Figure 6), and the best active phytochemical compound of quercetin observed occupying the active site in different ways can be seen in Figures 7, 8, 9, 10, 11, and Table 1.

### 3.9. MD

**Figure 12:**
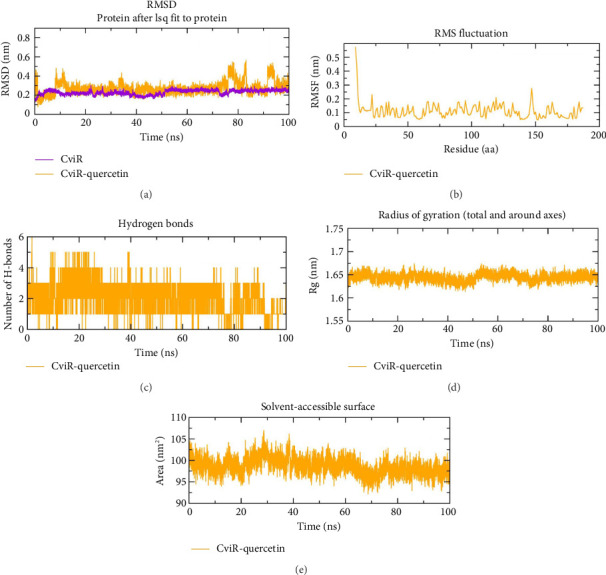
Molecular dynamics simulation analysis of the CviR protein and QC molecule over time. (a) RMSD analysis of CviR with and without QC binding, (b) RMSF analysis of the CviR–QC complex, (c) CviR–QC complex intramolecular H-bond time evolution, (d) Rg distribution of the CviR–QC complex, and (e) SASA plot analysis of the CviR–QC complex.

**Figure 13:**
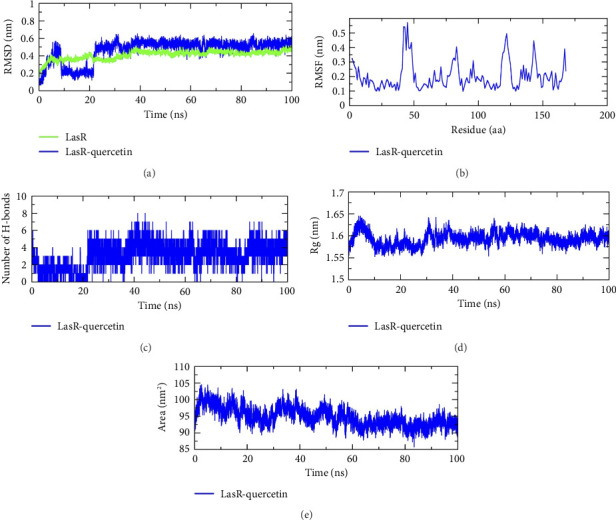
Molecular dynamics simulation analysis of the LasR protein and QC molecule over time. (a) RMSD analysis of LasR with and without QC binding, (b) RMSF analysis of the LasR–QC complex, (c) LasR–QC complex intramolecular H-bond time evolution, (d) Rg distribution of the LasR–QC complex, and (e) SASA plot analysis of the LasR–QC complex.

**Figure 14:**
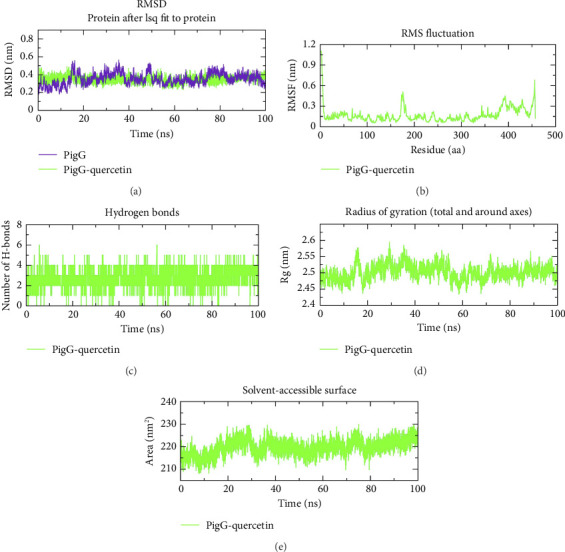
Molecular dynamics simulation analysis of the PigG protein and QC molecule over time. (a) RMSD analysis of PigG with and without QC binding, (b) RMSF analysis of the PigG–QC complex, (c) PigG–QC complex intramolecular H-bond time evolution, (d) Rg distribution of the PigG–QC complex, and (e) SASA plot analysis of the PigG–QC complex.

The MD simulation analysis was carried out for 100 ns to assess the behavior of QC with the top three proteins that showed strong binding affinity scores. The complexes of CviR, LasR, and PigG with QC were analyzed using the GROMACS package with an explicit TIP3P water model. In order to provide valuable insights into how small-molecule inhibitors interact with target proteins, MD simulations of the system providing information about the system's flexibility, stability, and the reliability of the binding mode are performed. The RMSD analysis was performed on the simulation trajectories in order to assess the stability of the ligand–protein complexes. RMSD measures how much a system deviates from its initial conformation over the course of the simulation, indicating system stability. For globular proteins, RMSD fluctuations within the range of 1–3 Å are generally considered acceptable. Fluctuations outside this range indicate significant conformational changes during the simulation. Detailed analysis of the trajectories for the three proteins bound to QC showed acceptable RMSD fluctuation patterns. The RMSD pattern was stable for CviR, and CviR–QC with averages of 0.24 nm and 0.26 nm (Figure 12(a)), respectively; for LasR and LasR–QC with averages of 0.43 nm, and 0.46 nm (Figure 13(a)); and for PigG and PigG–QC complexes with averages of 0.33 nm and 0.34 nm (Figure 14(a)). The CviR–QC complex exhibited minor fluctuations between 75-80 ns and 90–95 ns, while the LasR–QC complex showed slight fluctuations initially at 10–20 ns but eventually stabilized around the average value. The RMSF analysis was conducted to investigate the stability of amino acid residues in the active site, highlighting regional changes in the protein chain. The RMSF results for the CviR, LasR, and PigG complexes with QC showed minimal fluctuations, with average RMSF values of 0.11 nm (Figure 12(b)), 0.20 nm (Figure 13(b)), and 0.16 nm (Figure 14(b)), respectively. These values indicate enhanced stability of the protein chains and consistent interactions between the ligands and proteins. The relatively low RMSD values (all below 3 Å) and the minimal fluctuations seen in the RMSF plots further confirm the stability of the complexes. Based on an analysis of the MD trajectory data, it was evident that amino acid residues and simulated ligands were continuously interacting throughout the simulation. Hydrogen bonds play a critical role in maintaining the stability and integrity of protein structures. The time evolution of hydrogen bonds was calculated and plotted to assess the consistency of intramolecular bonds within the protein–ligand complexes. The intermolecular H-bonding formed within the CviR and CviR–QC, LasR and LasR–QC, and PigG, and PigG–QC docked complexes demonstrated stability. An average of four H-bonds was maintained between QC–CviR (Figure 12(c)), six for QC–LasR (Figure 13(c)), and four for QC–PigG (Figure 14(c)) complexes during the simulation. Although the number of H-bonds fluctuated at several points, an average of six H-bonds was maintained throughout the simulation for LasR–QC, four for CviR–QC, and four for PigG–QC. Additionally, the radius of gyration (rGyr) and SASA were also determined. The rGyr descriptor assesses the extent of a molecule's spread around its center of mass during the simulation. Higher rGyr values suggest increased flexibility and decreased stability of the molecule. The rGyr values for CviR–QC fluctuated between 1.62 and 1.66 Å, with an average of 1.64 Å (Figure 12(d)); for LasR–QC between 1.64 and 1.55 Å, with an average of 1.59 Å (Figure 13(d)); and for PigG–QC between 2.6 and 2.42 Å, with an average of 2.50 Å (Figure 14(d)). The SASA analysis provides insights into binding and protein folding of the ligand, with changes indicating the dynamics of the system and conformational changes. The average SASA values for CviR–QC, LasR–QC, and PigG–QC docked complexes were 98.61 (Figure 12(e)), 94.76 (Figure 13(e)), and 219.64 (Figure 14(e)) nm^2^, respectively.

## 4. Discussion

In recent years, bacteria have grown resistant to antibiotics, posing a serious health threat to the public [32, 50, 51]. During the past few years, there have been fewer discoveries of new efficient antibiotics, causing the situation to worsen in regard to the renewed ability of bacteria to develop resistance [52, 53]. It may be insufficient to use antibiotics despite their necessity, particularly when the infection is aggressively resistant [54–56]. Bacterial resistance can be diminished through the application of new approaches [57–60]. One of these approaches involves targeting bacterial virulence, which eases their eradication by immunity without stimulating bacteria to develop resistance [61, 62]. Bacterial QS systems play a crucial role in controlling bacterial virulence [63–65]. Further, the targeting of QS would have the effect of reducing several bacterial virulence factors like bacterial biofilm formation, enzyme production, the production of virulence agents, as well as motility [62, 66–68]. Many natural products and chemical compounds have been tested for their anti-QS and antivirulence properties [57, 69]. Plant-derived natural compounds are promising for advancing beyond traditional bacterial biofilm inhibition strategies. Among these, QC stands out due to its diverse biological activities, including notable antimicrobial properties. In recognition of its safety and efficacy, the FDA approved the use of high purity at doses up to 500 mg as a food ingredient in various categories in 2010 [70]. Building on this foundation, the current study investigates the anti-QS and antivirulence capabilities of quercetin against three Gram-negative bacteria, *C. violaceum S. marcescens*, and *P. aeruginosa*. This study aims to explore potential of quercetin to inhibit QS systems and reduce bacterial virulence, thereby offering a natural and effective approach to managing bacterial infections and mitigating resistance development.

*C. violaceum* is found predominantly in soil and water in tropical and subtropical regions. It is known for producing a characteristic violet pigment called violacein, which contributes to its distinctive coloration [71]. While *C. violaceum* is typically considered an environmental organism, it can be an opportunistic pathogen in humans and animals, capable of causing severe and sometimes fatal infections. In humans, infections with *C. violaceum* are rare but can lead to a variety of clinical manifestations. The bacterium can cause skin and soft tissue infections, which may present as cellulitis or abscesses following exposure to contaminated water or soil. More severe infections can occur, such as septicemia, which is characterized by the presence of bacteria in the bloodstream leading to widespread inflammation and multi-organ failure. Other potential complications include pneumonia, liver abscesses, and urinary tract infections. Due to its aggressive nature and rapid progression, *C. violaceum* infections require prompt medical intervention [72–74]. Additionally, this study utilized *S. marcescens* due to its key role in hospital-acquired infections. It has been documented as the seventh most common cause of nosocomial pneumonia and the 10th for bloodstream infections [33, 75, 76]. It also exhibits significant resistance to fluoroquinolones, aminoglycosides, and all β-lactams antibiotics, except cefepime and carbapenems due to the production of AmpC β-lactamase enzyme [37, 75, 76]. Moreover, to assess the antivirulence activity of QC, *P. aeruginosa* was also included in the study. *P. aeruginosa* is a significant hospital-acquired pathogen for its resistance to multiple antibiotics and its association with various infections in immunocompromised patients [77, 78]. Its array of virulence factors allows it to cause severe infections across various tissues [78, 79]. While the three selected Gram-negative bacteria for this study are clinically important, they each exhibit unique virulence behaviors and employ different QS systems.

An investigation of the inhibition of violacein production in *C. violaceum* was conducted to assess the anti-QS activity of QC. During the growth of *C. violaceum*, this pigment is produced under the control of the acylhomoserine lactone (AHL)-regulated QS. This assay measures anti-QS activity by measuring the reduction in pigment production. A spectrophotometric analysis was used to determine the quantitative content of this pigment. According to the results obtained in this study, QC treatment decreased pigment production in a concentration-dependent manner. The current results are similar to prior studies that found coumarins inhibited the synthesis of violacein in *C. violaceum* 12,472 [80]. *P. aeruginosa* produces the blue-green pigment pyocyanin through the action of QS. A dose-dependent decrease in pyocyanin levels in *P. aeruginosa* was observed in the presence of QC. Various cellular functions of the host are interfered with by pyocyanin, which contributes to *P. aeruginosa*'s pathogenicity. In individuals with cystic fibrosis, pyocyanin and its precursor have been observed to modify the expression of immune regulatory proteins and disrupt the regular movement of respiratory cilia [81]. In addition to assisting *P. aeruginosa* in formation of biofilm, pyocyanin inhibits the immune response of host by increasing neutrophil apoptosis [82]. Another pigment synthesized by *P. aeruginosa* is pyoverdine, which plays a crucial role in infection. By supplementing the culture media with QC, it was found that a concentration-dependent response was observed. Upon infection with *P. aeruginosa*, pyoverdine prevents transferrin from being absorbed into the host tissue, which results in iron deficiency [83]. This siderophore, which evades lipoplatin recognition, contributes to the development of *P. aeruginosa* infection during cystic fibrosis [84]. As a result, inhibition of these pigments demonstrates that QC can reduce pathogenicity of *P. aeruginosa*. As part of the biofilm structure, rhamnolipids are surfactants produced by *P. aeruginosa* that serve as adhesives which help bacterial cells to attach to surfaces, and which can also be utilized to maintain its biofilm structure [85]. A decrease in rhamnolipid production in *P. aeruginosa* was observed when QC was added to the culture. Additionally, these surfactants are also responsible for assisting *P. aeruginosa* in achieving surface motility. Previously, there was a study which showed that curcumin reduced the synthesis of rhamnolipids in *P. aeruginosa* PAO1 bacteria [86].

In *P. aeruginosa*, QS plays a crucial role in regulating bacterial motility by controlling the expression of genes involved in flagellar movement, twitching motility and biofilm formation, which are essential for surface attachment, colonization, and pathogenesis [87–90]. In order to further evaluate the anti-QS activity of QC, it was examined how it affected virulence factors controlled by QS in *S. marcescens*. The QS circuit of *S. marcescens* controls the production of prodigiosin, a red pigment. Approximately four AHLs are synthesized by *S. marcescens* and they are responsible for producing prodigiosin, forming biofilms and regulating motility. Additionally, QC treatment was shown to decrease prodigiosin production in a dose-dependent manner. In certain strains of *S. marcescens*, there might be a correlation between the production of prodigiosin, hemagglutination, and flagellar variation [91]. It is established that this bacterial pigment can suppress immune responses and induce cytotoxic effects in the host [92]. Bacteria produce pigments that are sometimes considered to be essential for their survival, as well as important for their pathogenic characteristics too [93]. The results of this study confirm those of an earlier study which found that petroselinic acid inhibited prodigiosin synthesis in *S. marcescens* ATCC 14756 [94].

Additionally, QC was evaluated in relation to the formation of biofilms by the three bacteria mentioned above. The formation of biofilms by the test bacteria was inhibited in a dose-dependent way. Most bacterial infections are associated with biofilms, which play a key role in the development of diseases [95]. Research has shown that pathogenicity of *P. aeruginosa* is attributed to biofilms, where bacterial cells exhibit increased resistance to physical and chemical interventions [96]. For instance, *P. aeruginosa* biofilms growing on urinary catheters are roughly a thousand times more resilient to tobramycin compared to freely suspended planktonic bacteria [97]. Previous studies have also noted that QC has the ability to hinder the formation of biofilms in various bacterial species such as, *Enterococcus faecalis* [98], methicillin- and vancomycin-resistant *Staphylococcus aureus* [99], *Staphylococcus saprophyticus* [99], *P. aeruginosa* [87–90], *C. violaceum* [87, 100], *Streptococcus mutans* [101], drug-resistant *Staphylococcus aureus* [100, 102], *Listeria monocytogenes* [103, 104], vancomycin-resistant *Enterococcus faecium* [105], *Bacillus subtilis* FB17 [106], *Streptococcus pneumoniae* D39 [107], *Escherichia coli* O157 [108], *Vibrio harveyi* [108], *Staphylococcus epidermidis* [109], Enteroaggregative *E. coli* strain 55,989 [110], *E. coli* K12 strain AR3110 [110], *Porphyromonas gingivalis* [111], and *S. marcescens* MG1 [112]. This study further confirms that QC inhibits the development of biofilms in Gram-negative bacteria on a broad spectrum. Moreover, inhibition of swarming motility of bacterial pathogen suggests that QC inhibits the rapid translocation of bacteria, which is crucial for efficient colonization on different types of surfaces.

Moreover, to explore the antivirulence potential of QC, computational studies were performed with proteins associated with QS and biofilm formation, revealing different binding affinities. CviR in *C. violaceum* senses QS signal molecules (C6-AHL) to activate QS-controlled genes which has ligand-binding and DNA-binding domains. CviR′ in *C. violaceum*, which shares 87% sequence similarity with CviR, senses 3-hydroxy-C10-AHL. Blocking the AHL binding site on these receptors can antagonize CviR activity, and QC binding may inhibit QS-linked traits. LasI in *P. aeruginosa* synthesizes 3-oxo-C12-HSL and shows homology with RhlI, another AHL synthase in *P. aeruginosa*. EsaI in *Pantoea stewartii* synthesizes 3-oxo-C6-HSL and also shares homology with RhlI. QC interaction with these AHL synthases might prevent precursor binding, inhibiting signal molecule synthesis. LasR, a transcriptional activator in *P. aeruginosa*, induces virulent genes upon binding 3-oxo-C12-AHL. QC may compete with 3-oxo-C12-AHL for LasR binding, reducing QS-controlled gene expression, as shown by QC inhibiting LasR-dependent traits like motility and biofilms. RhlR, another transcription regulator in *P. aeruginosa*, is activated by butanoyl-homoserine lactone. PqsR, a transcriptional regulator in *P. aeruginosa* is activated by *Pseudomonas* quinolone signal (PQS) and 2-heptyl-4-quinolone. PqsR controls major synthase genes in the pqsABCDE operon, and QC might inhibit AHL synthase synthesis. LasA in *P. aeruginosa*, involved in proteolytic and elastinolytic activities, is a significant virulence factor. Computational analysis also showed QC's interaction with PilT and PilY1, suggesting that QC's interaction with pilus proteins may inhibit biofilm formation.

## 5. Conclusion

The present study demonstrates the potent antibiofilm and anti-QS activities of QC against Gram-negative bacteria, highlighting its potential as a natural therapeutic agent. QC exhibited significant antibacterial effects, effectively inhibiting QS-mediated virulence factors, and biofilm formation in a dose-dependent manner at sub-MIC concentrations. QC notably reduced violacein and prodigiosin production in *C. violaceum* and *S. marcescens*, respectively, and significantly diminished the production of pyocyanin, pyoverdine, and rhamnolipids in *P. aeruginosa*. The reduction in biofilm formation was also significant across all tested bacteria along with inhibition of swimming motility and EPS production. Molecular docking and MD simulations revealed that QC interacts effectively with key QS and biofilm regulatory proteins, including LasI, LasR, PilY1, LasA, PilT, CviR, CviR′, PqsR, RhlR, and PigG. These findings suggest that the antibiofilm and anti-QS activities of QC are likely due to its interference with critical signaling molecules and regulatory proteins involved in these pathways. Overall, results of the present study highlight the potential of QC as a multifaceted antimicrobial agent, providing a foundation for its further exploration and development in antimicrobial therapies targeting biofilm-related infections caused by Gram-negative bacteria.

## Figures and Tables

**Table 1:** The residues of the target proteins that interact with QC in their best-fitting pose.

Sr. No.	Protein–compound	Receptor–lig,and	Interaction type	Distance
1	LasI–QC	A:ILE107:HN-UNL1:O	Conventional hydrogen bond	2.822
		A:THR144:OG1-UNL1:O	Conventional hydrogen bond	2.953
		UNL1:H-A:PHE105:O	Conventional hydrogen bond	2.808
		UNL1:H-A:ILE107:O	Conventional hydrogen bond	2.120
		A:ARG30:NH2-UNL1	Pi-cation; pi-donor hydrogen bond	3.420
		A:VAL26:CG1-UNL1	Pi-sigma	3.616
		A:VAL26:CG1-UNL1	Pi-sigma	3.924
		A:PHE105-UNL1	Pi-pi stacked	5.520
		A:TRP33-UNL1	Pi-pi T-shaped	5.953

2	PilY1–QC	A:SER732:HG-UNL1:O	Conventional hydrogen bond	2.269
		A:ARG848:HH1-UNL1:O	Conventional hydrogen bond	2.760
		A:ARG848:HH21-UNL1:O	Conventional hydrogen bond	2.812
		UNL1:H-A:SER732:O	Conventional hydrogen bond	2.580
		UNL1:H-A:PRO733:O	Conventional hydrogen bond	2.878
		UNL1:H-A:PRO733:O	Conventional hydrogen bond	2.783
		A:LYS790:NZ-UNL1	Pi-cation	4.471
		UNL1-A:VAL734	Pi-alkyl	5.248
		UNL1-A:PRO645	Pi-alkyl	5.317
		UNL1-A:VAL734	Pi-alkyl	4.532
		UNL1-A:LYS790	Pi-alkyl	5.350

3	LasA–QC	UNL1:H-A:GLY113:O	Conventional hydrogen bond	2.646
		A:TYR151:OH-UNL1	Pi-donor hydrogen bond	3.901
		A:TYR151:OH-UNL1	Pi-donor hydrogen bond	3.647
		UNL1:H-A:GLY113:O	Conventional hydrogen bond	2.646
		A:TYR151:OH-UNL1	Pi-donor hydrogen bond	3.901
		A:TYR151:OH-UNL1	Pi-donor hydrogen bond	3.647
		A:TYR80-UNL1	Pi-pi T-shaped	5.494

4	CviR′–QC	A:ASP97:OD2-UNL1	Pi-anion	3.953
		A:LEU72:CD1-UNL1	Pi-sigma	3.535
		A:TYR88-UNL1	Pi-pi stacked	3.749
		A:TYR88-UNL1	Pi-pi stacked	4.733
		UNL1-A:LEU57	Pi-alkyl	5.281
		UNL1-A:LEU57	Pi-alkyl	5.175

5	CviR–QC	A:SER155:HG-UNL1:O	Conventional hydrogen bond	2.285
		UNL1:H-A:MET89:SD	Conventional hydrogen bond	2.284
		UNL1:H-:UNL1:O	Conventional hydrogen bond	1.846
		A:ASP97:OD2-UNL1	Pi-anion	3.923
		A:ASP97:OD2-UNL1	Pi-anion	3.587
		A:TYR80:OH-UNL1	Pi-donor hydrogen bond	3.659
		A:TYR88-UNL1	Pi-pi stacked	4.705
		A:TYR88-UNL1	Pi-pi stacked	4.475
		A:TYR80-UNL1	Pi-pi T-shaped	5.483
		A:TYR80-UNL1	Pi-pi T-shaped	5.043
		UNL1-A:LEU57	Pi-alkyl	5.020
		UNL1-A:ILE99	Pi-alkyl	5.386
		UNL1-A:LEU72	Pi-alkyl	5.243
		UNL1-A:VAL75	Pi-alkyl	4.187

6	LasR–QC	G:ARG61:NH2-UNL1:O	Conventional hydrogen bond	2.811
		G:THR75:OG1-UNL1:O	Conventional hydrogen bond	2.942
		G:THR115:HG1-UNL1:O	Conventional hydrogen bond	2.743
		G:SER129:OG-UNL1:O	Conventional hydrogen bond	2.799
		UNL1:H-G:LEU125:O	Conventional hydrogen bond	2.461
		UNL1:H-G:LEU125:O	Conventional hydrogen bond	2.199
		UNL1:H-:UNL1:O	Conventional hydrogen bond	1.767
		G:ASP73:OD2-UNL1	Pi-anion	3.728
		G:LEU36:CD2-UNL1	Pi-sigma	3.770
		G:TYR64-UNL1	Pi-pi stacked	5.163
		G:TYR64-UNL1	Pi-pi stacked	5.056
		UNL1-G:VAL76	Pi-alkyl	4.995
		UNL1-G:ALA127	Pi-alkyl	4.732
		UNL1-G:LEU36	Pi-alkyl	4.479
		UNL1-G:ALA127	Pi-alkyl	5.201
		UNL1-G:LEU40	Pi-alkyl	5.431
		UNL1-G:ALA50	Pi-alkyl	5.224
		UNL1-G:VAL76	Pi-alkyl	4.893
		UNL1-G:ALA127	Pi-alkyl	4.660

7	PqsR–QC	UNL1:H-A:LEU208:O	Conventional hydrogen bond	2.141
		A:LEU208:CD2-UNL1	Pi-sigma	3.754
		A:ILE236:CG2-UNL1	Pi-sigma	3.550
		UNL1-A:LEU208	Pi-alkyl	5.070
		UNL1-A:ILE236	Pi-alkyl	4.596
		UNL1-A:ILE236	Pi-alkyl	4.962
		UNL1-A:ILE263	Pi-alkyl	4.578

8	PigG–QC	A:LEU264:HN-UNL1:O	Conventional hydrogen bond	2.470
		UNL1:H-A:GLY262:O	Conventional hydrogen bond	2.105
		A:LYS17:NZ-UNL1	Pi-cation	3.905
		A:GLU113:OE1-UNL1	Pi-anion	3.566
		A:GLU113:OE2-UNL1	Pi-anion	3.745
		UNL1-A:LEU264	Pi-alkyl	4.966

9	RhiR–QC	D:VAL47:HN-UNL1:O	Conventional hydrogen bond	2.668
		D:TRP68:HE1-UNL1:O	Conventional hydrogen bond	2.053
		D:TRP68:HE1-UNL1:O	Conventional hydrogen bond	2.084
		D:ASP81:OD2-UNL1	Pi-anion	3.233
		D:ASP81:OD2-UNL1	Pi-anion	3.634
		D:VAL60:CG2-UNL1	Pi-sigma	3.629
		D:ILE84:CD-UNL1	Pi-sigma	3.239
		D:TYR72-UNL1	Pi-pi stacked	4.433
		D:TRP96-UNL1	Pi-pi stacked	5.283
		UNL1-D:ALA83	Pi-alkyl	5.238

10	PilT–QC	A:THR139:OG1-UNL1:O	Conventional hydrogen bond	2.997
		A:ARG276:NE-UNL1:O	Conventional hydrogen bond	2.928
		A:ARG276:HH12-UNL1:O	Conventional hydrogen bond	2.166
		UNL1:H-A:LEU109:O	Conventional hydrogen bond	2.516
		UNL1:H-A:GLY135:O	Conventional hydrogen bond	2.504
		UNL1:H-UNL1:O	Conventional hydrogen bond	1.832
		A:GLY135:CA-UNL1:O	Carbon hydrogen bond	3.276
		A:LEU109:CD2-UNL1	Pi-sigma	3.917
		A:LEU109:CD2-UNL1	Pi-sigma	3.678
		A:LEU268:CD2-UNL1	Pi-sigma	3.945
		A:ALA278:CB-UNL1	Pi-sigma	3.424
		UNL1-A:ALA278	Pi-alkyl	4.641
		UNL1-A:ARG276	Pi-alkyl	5.330

## Data Availability

The information supporting this study is available in this article.
